# Probiotics use for antibiotic-associated diarrhea: a pragmatic participatory evaluation in nursing homes

**DOI:** 10.1186/s12876-020-01297-w

**Published:** 2020-05-13

**Authors:** Herman A. van Wietmarschen, Martine Busch, Annemiek van Oostveen, Gerda Pot, Miek C. Jong

**Affiliations:** 1 Department Nutrition & Health, Louis Bolk Institute, Kosterijland 3-5, 3981 Bunnik, AJ The Netherlands; 2 Van Praag Institute, Springweg 7, 3511 Utrecht, VH The Netherlands; 3 Rivas Zorggroep, Sliedrecht, The Netherlands; 4 Department of Health Sciences, Mid Sweden University, Holmgatan 10, 851 70 Sundsvall, Sweden

**Keywords:** Probiotics, AAD, Antibiotic use, Elderly, Somatic conditions, Psychogeriatric conditions, Urinary tract infection

## Abstract

**Background:**

Antibiotic-associated diarrhea (AAD) occurs in 2–25% of nursing home residents, which may lead to dehydration, malnutrition, severe complications and hospitalizations. Research shows that probiotics can be effective and safe in reducing AAD. However, probiotics are not routinely used in Dutch nursing homes. The objectives of this evaluation were to develop a procedure for the implementation of probiotics to prevent AAD in nursing homes, to evaluate effects on AAD occurrence, and to evaluate the implementation process of probiotics in daily care.

**Methods:**

A pragmatic participatory evaluation (PPE) design was chosen, as it seemed a suitable approach for implementation of probiotics, as well as for evaluation of its effectiveness in daily nursing home practice. Probiotics administration was implemented in three nursing homes of the Rivas Zorggroep for residents with somatic and/or psychogeriatric conditions. Ninety-three residents provided data on 167 episodes of antibiotics use, of which 84 episodes that included supplementation with probiotics and 83 episodes with no probiotics supplementation. **A** multispecies probiotics was administered twice daily upon start of antibiotic treatment, up to 1 week after completing the antibiotics course. The occurrence of AAD was monitored and a process evaluation was conducted to assess facilitators and barriers of probiotics implementation.

**Results:**

The number of episodes with AAD when using probiotics was significantly lower than when no probiotics was used (20% vs 36%; *p* = 0,022, Chi-square). No significant differences in the occurrence of AAD were found between the residents taking amoxicillin/clavulanic acid or ciprofloxacin. Reported facilitators for implementation were perceived benefits of probiotics and prescription by medical staff. Reported challenges were probiotics intake by residents and individual decision-making as to which resident would benefit from it.

**Conclusion:**

Successful implementation of probiotics demonstrated the prevention of AAD in nursing home residents.

**Trial registration:**

ISRCTN 94786163, retrospectively registered on 3 February 2020.

## Background

Up to 10% of nursing home residents will be on antibiotics at any moment in time [1]. The most common indication for antibiotics use in nursing homes is urinary tract infection, followed by lower respiratory tract infection and skin infection [2]. Antibiotic-associated diarrhea (AAD) occurs in 2–25% of nursing home residents depending on the type of antibiotic prescribed [3]. Symptoms often appear a few days after starting antibiotics, and can last up to 10 weeks [4]. AAD in nursing homes can lead to severe complications resulting in dehydration and malnutrition as well as hospital admissions thus leading to extra healthcare costs [1]. Furthermore, antibiotics use increases the risk of developing a *Clostridium difficile* infection, which often leads to discontinuation of antibiotics, and requires strict hygiene measures to control the infection [5]. Therefore, there is a high need for effective interventions that can reduce AAD.

Probiotics are ‘live microorganisms that when administered in adequate amounts confer a health benefit on the host’ [6]. Several blends of multispecies probiotics, as well as Lactobacillus, Enterococcus and Saccharomyces species appear to be safe and effective in reducing AAD overall [7], as shown in outpatients [8], as well as free-living adults and children [9]. No serious adverse events have been found to be attributed to probiotics, and the risk for adverse events has been similar to that of control groups [8, 9]. Systematic reviews and meta-analyses show potential benefits for the use of both multispecies and mono-species probiotics in reducing AAD [10, 11]. Overall, it appears that probiotic use may be beneficial in the prevention of AAD in all age groups, although not all types of probiotics seem to be equally effective in preventing AAD in older populations [12]. The question that remains to be answered is how the effectiveness of probiotics, as demonstrated in clinical trials, translates into daily nursing home practice. Practical and organizational contextual factors, such as how and when to administer probiotics to prevent AAD, related costs, and attitudes of healthcare staff towards probiotics, need to be considered for successful implementation [1]. Therefore, the present evaluation of the implementation of probiotics in Dutch nursing homes was initiated.

The aim of this evaluation was twofold: 1) evaluation of changes in the occurrence of AAD by administrating multispecies probiotics to nursing home residents, and 2) evaluation of the implementation process of probiotics in daily nursing home practice.

## Methods

### Design

**Fig. 1:**
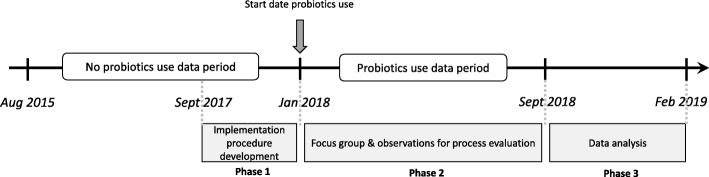
Timeline of the pragmatic participatory evaluation consisting of three phases and two time periods from which data is collected

A pragmatic participatory evaluation (PPE) design was chosen as a suitable approach for implementation of probiotics, as well as to study its effectiveness in daily nursing home practice. The PPE design included active engagement of healthcare staff in each stage of the process [13]. Participatory action research supports a pragmatic design that includes outcomes relevant for daily nursing home practice [14, 15]. The PPE took place from September 2017 to February 2019 and was carried out by a project team that consisted of an elderly care physician, dietician and pharmacist of the Rivas Zorggroep, a researcher from the Louis Bolk Institute (Bunnik) and a process manager from the van Praag Institute (Utrecht). The PPE consisted of three phases in time: phase 1) development of the implementation procedure, phase 2) collection of data for outcome and process evaluation, phase 3) data analysis and interpretation. All members of the project team participated in the development of the implementation procedure, design of the evaluation, collection of data, and process evaluation. Data was obtained from patient records retrospectively (see Fig. 1).

### Setting

Rivas Zorggroep is an organisation that provides various types of care facilities for over 1000 residents in a total of 18 locations throughout the Netherlands. Probiotics administration was implemented in three nursing homes of the Rivas Zorggroep for residents with somatic and/or psychogeriatric conditions: Alblashof in Alblasserdam (60 rooms), Waalburcht in Papendrecht (75 rooms), and Waerthove in Sliedrecht (150 rooms).

### Participants

Participants in this PPE were residents with somatic and/or psychogeriatric conditions of three nursing homes from the Rivas Zorggroep. All residents requiring amoxicillin/clavulanic acid or ciprofloxacin were allowed to participate after giving consent.

### Implementation of probiotics

The probiotic that were implemented and evaluated in the three nursing homes was the multispecies probiotics Ecologic® AAD (Winclove Probiotics B.V.) [16]. Ecologic AAD consists of 9 different bacterial species, the total daily dose being 10^10^ cfu (*Bifidobacterium bifidum* W23, *Bifibacterium longum* W51, *Enterococcus faecium* W54, *Lactobacillus acidophilus* W37 and W55, *Lactobacillus paracasei* W20, *Lactobacillus plantarum* W62, *Lactobacillus rhamnosus* W71, and *Lactobacillus salivarius* W24). A protocol was developed for the nursing staff with instructions about the probiotic administration procedure, who it should be administered to, with which food substances it could be mixed, and instructions about special situations such as problems with swallowing, or probe feeding. Information on the use and administration of probiotics was obtained from the instruction for use issued by the probiotic manufacturer, as well as from a previous publication [16]. Although probiotics are usually considered dietary supplements, it was decided that the elderly care physicians should prescribe probiotics as standard additional treatment to the antibiotic prescription. Therefore, probiotics were listed on the medication list of the respective nursing home resident. The medication list is used by the nursing staff to keep track of all given medication and requires that the staff checks off the use of both antibiotics and probiotics, which guarantees administration of the probiotics. Based on previous publications [17] as well as the most frequently used antibiotics at Rivas zorggroep, amoxicillin/clavulanic acid and ciprofloxacin were selected for which concomitant probiotics intake was considered useful. Therefore, all residents requiring antibiotics amoxicillin/clavulanic acid or ciprofloxacin received probiotics as part of standard care, except in specific cases were residents were not physically able to consume the probiotics. The protocol prescribed that for each administration one 5 g sachet of probiotics should be mixed with 100 ml water, milk or (drink) yoghurt by the nursing staff. Mixing with fruit juice, fruit compote, soft drinks or warm drinks was not allowed. The probiotics were administered twice daily, at least two to 3 hours before or after the administration of antibiotics to prevent inhibition of the probiotic bacterial species by the antibiotics [18]. The probiotics were given to residents at the start of an antibiotics treatment until 1 week after finishing the treatment [19]. The dietician was designated as the main contact person for questions regarding the protocol and administration of probiotics and monitored the actual administration of probiotics together with the nursing staff.

### Outcome evaluation

For the outcome evaluation, data were collected from the medical records of residents that used probiotics in the period January 2018 till the end of August 2018. Additionally, control data from episodes of antibiotics (amoxicillin/clavulanic acid or ciprofloxacin) use without probiotics were collected from both medical records of the same residents that later used probiotics as well as from other residents that required antibiotics up to 27 months prior to the evaluation period (Fig. 1). Therefore, data from multiple episodes of antibiotics use per resident were available. Changes in residents’ stools were routinely recorded in the medical records of the residents by nursing staff. Antibiotics associated diarrhoea was defined as stools described as looser than normal stools during a period of a few weeks before antibiotics use. The elderly care physician extracted data from episodes of antibiotic use with and without probiotics from the medical records and judged whether the diarrhoea occurred as a result of each of the episodes of antibiotics use, in which case it was defined as AAD. The elderly care physician was not blinded for who received probiotics and who not. The start and end dates of the administration of probiotics and antibiotics, the indication for the antibiotics, the location of the resident, the type of antibiotics, the age and gender of the residents, and occurrence of adverse events were recorded. The occurrence of AAD was calculated by comparing episodes of antibiotic use with or without probiotics supplementation.

### Statistical analysis

Descriptive analyses were conducted using SPSS, version 24. The incidence of AAD in the episodes with probiotics and the ones without probiotics were compared using the Chi-square test. The incidence of AAD was also calculated per type of infection (classified as urinary tract infection, respiratory tract infection, and other infections) and per type of antibiotics (amoxicillin/clavulanic acid or ciprofloxacin) used. Additionally, a pre-specified subgroup analysis was conducted with data from residents that provided data from an episode of antibiotic use with probiotics and without probiotics. Data is available in supplementary file S2 Data.

### Process evaluation

In participatory action research, a wide range of data collection methods are used, including observations and focus groups [20]. Focus group are frequently used in participatory research because it helps to create a communicative space in which open dialogue can take place. They are also used to collect data and validate findings [20]. During two project team meetings (in September 2017 and January 2018) and three focus groups (two in June 2018 and one in Oktober 2018), detailed observations and notes were written down by one of the researchers for the purpose of process evaluation. The three focus groups of 2 hours were conducted with six to eight participants. Focus groups were conducted to collect experiences, needs and expectations of the nursing staff, dietician, two elderly care physicians, and a clinical nurse specialist regarding the use of probiotics in their nursing home locations. The focus groups were structured along four topics: 1) information provided to the nursing staff, 2) administration of probiotics, 3) observed effects of probiotics, 4) the future of probiotics use. Focus group discussions were recorded and field notes were taken. Written outcome of the focus groups was sent to the participants for correctness and feedback and/or additions were taken into account. The content of the focus groups were thematically analysed focussing on the facilitators, challenges and protocol adaptations for implementation of probiotics.

## Results

### Outcome evaluation

**Fig. 2:**
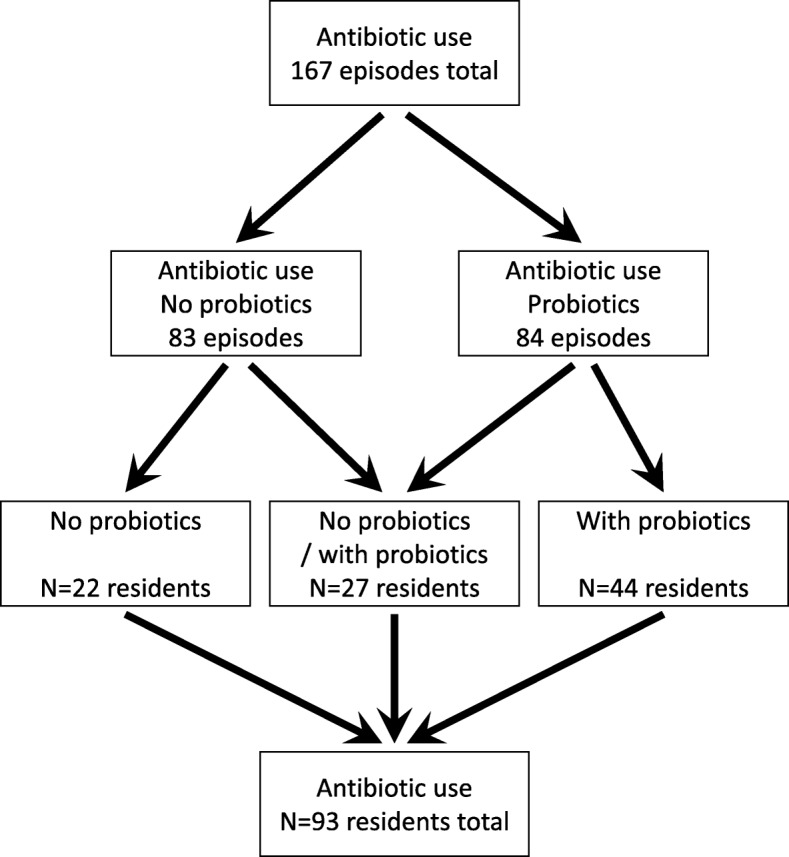
Relationship between data collection of number of residents and episodes of antibiotics use

As shown in Fig. 2, data on 167 episodes of antibiotics use were collected from a total of 93 residents, including 84 episodes of antibiotics use that were supplemented with probiotics and 83 episodes of antibiotics use with no probiotics supplementation (see S2 Data). Data from the 83 episodes on antibiotics use with no probiotics were derived from 49 residents. Data from the 84 episodes on antibiotics use with probiotics use were derived from 71 residents. Thus, 27 residents had episodes of antibiotic use both with and without probiotics use (Fig. 2). Furthermore, in 45% of residents, data were collected on more than one episode of antibiotics use. No serious adverse events were reported and adverse events were observed in two out of 71 residents (2,8%) upon probiotics use. The reported adverse events were nausea and a bloated feeling. In both residents, the occurrence of adverse events led to discontinuation of probiotic intake. Since both residents reported intestinal complaints before starting the probiotics, a causal relationship with probiotic intake was evaluated as unlikely. Data of these two residents were not included in the evaluation.

Characteristics of residents and antibiotic use are shown in Table 1. Both data sets, no probiotics versus probiotics, were comparable with respect to the age and gender of residents. No significant differences were found between the episodes of antibiotic use with and without probiotics. Antibiotics were prescribed in the majority of cases for urinary tract- and respiratory tract infections (Table 1). Other infections were skin infection (*N* = 8), wound infection (*N* = 3), bowel perforation (*N* = 2), otitis media (*N* = 1), cellulitis (*N* = 1), erysipelas (*N* = 1), and tooth infection (*N* = 1). The average duration of antibiotics treatment was 8 days, and the average duration of probiotics treatment was almost 15 days (Table 1).

**Table 1:** Characteristics of residents and episodes of antibiotic use

Characteristic	Value	All	No probiotics	Probiotics
Number of residents	N	93	49	71
Age	Year (mean (SD))	84 (10,0)	83 (10,7)	85 (9,6)
Male	N (%)	36 (30%)	14 (29%)	22 (31%)
Female	N (%)	82 (70%)	35 (71%)	48 (69%)
Number of antibiotic episodes of residents	N	167	83	84
Type of antibiotic used				
amoxicillin/clavulanic acid	N (%)	83 (50%)	44 (53%)	39 (46%)
ciprofloxacin	N (%)	84 (50%)	39 (47%)	45 (54%)
Indication antibiotic prescription)				
urinary tract infection	N (%)	99 (59%)	45 (54%)	54 (64%)
respiratory tract infection	N (%)	51 (31%)	24 (29%)	27 (32%)
other	N (%)	17 (10%)	14 (17%)	3 (4%)
Duration antibiotics	Days (mean (SD))	8,2 (2,8)	8,4 (3,0)	8,0 (2,6)
Duration probiotics	Days (mean (SD))			14,7 (3,9)

The occurrence of AAD incidence in episodes of antibiotic use with and without probiotic supplementation is reported in Table 2. The number of episodes of antibiotic use with AAD when using probiotics was significantly lower than when no probiotics were used (20% vs 36%; *p* = 0,022). No significant difference in the frequency of AAD occurrence was found between the two types of antibiotics used (Table 2).

**Table 2:** Frequencies of AAD incidence in episodes of antibiotics use with and without probiotic supplementation

	Value	No probiotics		Probiotics	
		No AAD	AAD	No AAD	AAD
**Between groups**					
Episodes	N (%)	53 (64%)	30 (36%)	67 (80%)	17 (20%)^a^
**Within groups**	N	53	30	67	17
Type of antibiotic used					
amoxicillin/clavulanic acid	N (%)	27 (51%)	17 (57%)	30 (45%)	9 (53%)
ciprofloxacin	N (%)	26 (49%)	13 (43%)	37 (55%)	8 (47%)
Indication antibiotic prescription					
urinary tract infection	N (%)	31 (58%)	14 (47%)	45 (67%)	9 (53%)
respiratory tract infection	N (%)	13 (25%)	11 (37%)	19 (28%)	8 (47%)
other	N (%)	9 (17%)	5 (17%)	3 (4%)	0 (0%)

^a^Significant difference in AAD occurrence between no probiotics and probiotics group, *p* = 0,022, Chi-square

From 27 residents data were collected from both episodes of antibiotic use with and without probiotics supplementation. In 27 residents with multiple antibiotic episodes, AAD occurred in 15% (*n* = 4) of the residents when on probiotics compared to 52% (*n* = 14) of the residents when antibiotics were given without probiotics. One of the 13 residents not developing AAD during antibiotics treatment without probiotics actually developed AAD when using probiotics during a later episode of antibiotics use. 3 (15%) of the 14 residents which did develop AAD during antibiotics treatment without probiotics, did also develop AAD when taking antibiotics in combination with probiotics treatment.

### Process evaluation

The process evaluation was based on observations during two project team meetings and three focus group sessions. In total 13 nurses or caretakers, a clinical nurse specialist, two elderly care physicians and one dietician joined in at least one of the focus group sessions. Results from the process evaluation were thematically categorized in facilitators, challenges and protocol adaptations for implementation of probiotics in Dutch nursing homes.

#### Facilitators

Several facilitators for the implementation process were identified. The use of probiotics fits within the current societal trend of healthy lifestyle and prevention. Nursing home residents were in general curious, open for improvements and had supporting families. Nursing staff perceived the benefits of probiotics: They were of the opinion that it was important to contribute to less AAD, to improve residents’ quality of life and to save time washing residents and changing diapers. The presence of the dietician as the designated contact person for nursing staff to answer practical questions about logistics, was also found to be a facilitator as it motivated the nursing staff. A team of eight elderly care physicians prescribed the probiotics and supported the implementation, with strong leadership from one elderly care physician that mobilized support from colleagues, was able to involve others (management, nurses, resident council), and prioritized and invested time in the implementation process. Finally, Rivas Zorggroep management facilitated implementation by allowing the probiotics to be administered through the medication registration system, and an in-house pharmacist that took responsibility for ordering, distribution and storage of the probiotics at the various locations.

#### Challenges

One of the challenges for the nursing staff was that residents had to consume the probiotics. Many residents did not want to drink in the afternoon out of fear for nycturia. Probiotics were mostly mixed with water by nursing staff, while it was also allowed to mix these with dairy products. Furthermore, not all residents liked the taste of the probiotics in water. It was a challenge for the dietician to break the habit of nurses to mix the probiotics with water and convince them to choose more tasty options. Furthermore, administration of probiotics required extra time from nursing staff, especially because it was not allowed to administer it at the same time as the antibiotics. Another challenge was to take the individual situation of each resident into account as to decide for each whether the use of probiotics was a beneficial option or not.

#### Protocol adaptations

Adaptations to the developed implementation protocol were made, in order to provide better instructions to the nursing staff on how to administer the probiotics to nursing home residents. Nursing staff required specific information whether probiotics could be combined with laxatives, whether probiotics had possible interactions with other medication, and whether probiotics could be administered to residents with gluten intolerance. Additionally, the nursing staff required recommendations on administering probiotics in a more individualized manner to residents. For example, adjusting the dosage and starting moment of the probiotics, or changing the administration time (earlier or later on the day). All feedback was taken into account in the development of a final version of the implementation protocol (see Additional file 1)

## Discussion

In this pragmatic participatory evaluation, the feasibility to implement probiotics for the prevention of AAD in daily nursing home practice is demonstrated. The implementation process required cooperation from all healthcare staff, including a dietician and pharmacist. In order to make sure that probiotics are administered effectively, it was necessary to have it prescribed by the elderly care physicians of the nursing home, and to add it to the medication list, even though probiotics is a dietary supplement. The efficacy of the specific multispecies probiotic that was implemented in the current PPE, has not previously been investigated in the prevention of AAD in nursing home residents, but was tested in healthy volunteers taking amoxicillin [16]. No differences were observed in bacterial counts and metabolic activity between the probiotic versus placebo group. However, a small but significant reduction in diarrhea-like bowel movements was observed in the probiotic group, suggesting that this multispecies is likely to decrease diarrhea-like defecation [16]. The present PPE showed that this multispecies probiotics significantly reduced AAD in nursing home residents. These findings are in line with earlier studies that demonstrated reduction of AAD upon administration of probiotics containing *Lactobacillus acidophilus* in older populations [21, 22]. A recent meta-analysis confirms the potential of probiotics mixtures including *L. acidophilus* for the prevention of AAD, while mixtures with *L. rhamnosus* was not shown effective [23]. A previous study showed no difference between multispecies probiotics (*L. acidophilus, B. bifidum, B. lactis*) versus placebo in the prevention of AAD in older (≥65 years) hospital patients [3]. However, probiotics were administered up to 7 days after the start of the antibiotics treatment, and not always at the start of antibiotics treatment as is the case in the present PPE. This may explain the lack of effect in the study by Allen et al. Furthermore, in the present study the administration of probiotics to nursing home residents with somatic and/or psychogeriatric conditions was found to be safe. Adverse events where only reported in 2.8% of cases, and were not likely to be caused by probiotics intake and no serious adverse events were reported. A previous study indicates that the use of *L. rhamnosus* is safe in older populations [24], but that probiotic treatment is contraindicated for critically ill patients with an immune compromised state, an impaired intestinal barrier function such as occurs with multi organ failure and severe acute pancreatitis, and a central venous catheter [25].

This PPE design had several limitations that need further discussion and reflection. The protocol for implementation of probiotics for prevention of AAD in older people was specifically developed for the nursing home practice of the Rivas Zorggroep in the Netherlands. The protocol was only evaluated in three nursing homes, of which two were relatively small. Therefore, the outcome of the present process evaluation may not be representative for other nursing homes in the Netherlands. It is recommended that other long-term care facilities that want to implement probiotics, adapt the protocol according to their own internal organisation and context, and take into account the facilitators and barriers as experienced in this PPE. Another limitation is that a convenience sample of nursing home residents was used to evaluate the changes in AAD, comparing a group with probiotics to a group with no probiotics. Data for comparison were collected retrospectively (no probiotics) and prospectively (probiotics), and for 27 out of 93 residents comparison data derived from the same person. The total number of eligible residents that were on amoxicillin/clavulanic acid during the period of the study, and the number of those eligible residents receiving probiotics was not registered. A certain degree of selection bias may thus have occurred and the observed significant differences in occurrence of AAD incidence with or without probiotics should therefore be interpreted with caution. Furthermore, residents were not randomly allocated to the two groups. The observed significant differences in occurrence of AAD might therefore also be due to other unknown factors rather than to probiotics supplementation. Another limitation of the present PPE was that delayed-onset AAD and possible effects of probiotics on the frequency of delayed-onset of AAD were not monitored. Finally, a standardized method such as the Bristol stool form chart for classification of the form of stool and blinding of the elderly care physician for probiotic use, might have resulted in more objective assessment of the stool consistency.

The large diversity of probiotics that are available on the market, makes it difficult for healthcare professionals and consumers to decide which probiotics to choose for the prevention of AAD. Up till now, probiotics are not considered in any European guidelines for prevention of AAD in older people [26]. In order to support decision-making of healthcare professionals on the selection of probiotics, practical guides have been published recently in the scientific literature [17, 27]. Eight different probiotic products in the Netherlands are recommended for the prevention of AAD according to the practical guide for probiotics applied to the case of AAD in the Netherlands, among them the multispecies probiotic as implemented in the present study [17].

Since the present PPE demonstrated that the incidence of AAD in nursing home residents can be reduced upon probiotics supplementation, it is of interest to investigate whether the use of probiotics is cost-effective. A previously published study estimated that probiotics use for the prevention of AAD leads to significant savings in direct medical costs of hospitalized patients [22]. Cost-related outcome measures were not evaluated in the present PPE, but its outcome warrants further studies into the cost-effectiveness of probiotics. Another interesting subject for further research is whether this multispecies probiotics can prevent recurrent urinary tract and other infections that occur in older people, since that objective was not included in the present study.

## Conclusions

In conclusion, successful implementation of probiotics demonstrated a reduction in the occurrence of AAD in nursing home residents. Further studies are recommended to investigate whether probiotic use for prevention of AAD is cost-effective and whether it can prevent recurrent infections in older people.

## Supplementary information

**Additional file 1.** Probiotics Administration Protocol

**Additional file 2.** Raw data of the probiotics evaluation

## Data Availability

The raw data used to produce the results presented in this manuscript is available as a supplementary file (S2 Data).

## Abbreviations

AAD: Antibiotic-associated Diarrhea
BV: Besloten Vennootschap, English Ltd. or American LCC
PPE: Pragmatic Participatory Evaluation
SD: Standard Deviation
SPSS: Statistical Package for the Social Sciences
WMO: Dutch Medical Research in Human Subjects Act (Wet Medisch-wetenschappelijk Onderzoek)
