# Supplementary material for: Probiotics use for antibiotic-associated diarrhea: a pragmatic participatory evaluation in nursing homes
Source: BMC Gastroenterol. 2020 May 13;20:151. doi: 10.1186/s12876-020-01297-w (PMC7222499; doi:10.1186/s12876-020-01297-w)
Supplement: Supplementary file 1 — Additional file 1. Probiotics Administration Protocol [file 12876_2020_1297_MOESM1_ESM.docx]

Supplementary Text S1 Probiotics Administration Protocol

The Probiotics Administration Protocol below is an updated version of the protocol used by Rivas Zorggroep based on the focus group discussions.

| **Administration of probiotics (powder form)** |
| --- |
| Aim  Practical guidelines for correct administration of probiotics  Target group  This document is meant for nursing staff working in nursing homes.  Materials   - One 5 g sachet of probiotics (powder form) - 100 ml water, dessert, milk or (drink)yoghurt   Description of the administration procedure  **General considerations**   - Mix the powder with 100 ml water, dessert, milk or (drink)yoghurt. Don’t mix it with fruit juice, fruit compote, carbonized drinks or warm drinks. Stir well before use. - Administer probiotics at least 2-3 hours before or after the administration of antibiotics. - Administer probiotics twice per day from the start of antibiotics treatment up to 1 week after finishing the antibiotics treatment.   **In case of swallowing problems**   - Mix probiotics with thickener, dessert or pudding. Don’t thicken with fruit compote and/or warm foods.   **In case of probe feeding**   - Mix the powder with 100 ml water. Stir well before administration by probe. Don’t administer probiotics when the probe is positioned past the stomach.   **In case of diarrhoea**   - Probiotics administration is allowed in cases of diarrhoea.   **In case of lactose intolerance**   - Probiotics can be used in cases of lactose intolerance.   **In case of laxative use**   - Probiotics administration is allowed in conjunction with laxatives. |
